# How zoledronic acid improves osteoporosis by acting on osteoclasts

**DOI:** 10.3389/fphar.2022.961941

**Published:** 2022-08-25

**Authors:** Biao Wang, Yi Zhan, Liang Yan, Dingjun Hao

**Affiliations:** ^1^ Spine Surgery, Honghui Hospital Affiliated to Xi’an Jiaotong University, Xi’an, China; ^2^ The Sceond Clinical Medical College of Shaanxi University of Chinese Medicine, Xi’an, China

**Keywords:** osteoporosis, zoledronic acid, osteoclasts, differentiation, apoptosis, signaling, signaling pathways

## Abstract

Osteoporosis is called a silent disease, because it is difficult to detect until comprehensive examinations for osteoporosis are performed or osteoporotic fractures occur. Zoledronic acid is currently the first-line anti-osteoporotic drug, with good efficacy and treatment compliance. A major advantage of zoledronic acid is that intravenous zoledronic acid often guarantees a therapeutic effect for up to 1 year after infusion. The reasons why zoledronic acid is effective in improving osteoporosis are that it can inhibit osteoclast differentiation and induce osteoclast apoptosis, thus suppressing bone resorption and increasing bone density. The story between zoledronic acid and osteoclasts has been written long time ago. Both the canonical receptor activator of the receptor activator of nuclear factor-κB ligand (RANKL) pathway and the non-canonical Wnt pathway are the main pathways by which zoledronic acid inhibits osteoclast differentiation. Farnesyl pyrophosphate synthase (FPPS), reactive oxygen species (ROS), and ferroptosis that was first proposed in 2012, are all considered to be closely associated with zoledronic acid-induced osteoclast apoptosis. Here, we provide a brief review of the recent progress on the study of zoledronic acid and osteoclasts, and hope to elaborate how zoledronic acid improves osteoporosis by acting on osteoclasts.

## Introduction

Osteoporosis is a growing social problem, that can impact the quality of life in elderly people not only medically, but also socially and economically (Rachner et al., 2011; Camacho et al., 2020). Osteoporosis is a highly prevalent disease, and is estimated to affect more than 200 million people over the age of 60 worldwide. Globally, 1 in 3 women and 1 in 5 men, over the age of 50, will experience osteoporotic fractures in their lifetime, and it is estimated that osteoporosis causes more than 8.9 million fractures annually (Wang et al., 2021). Osteoporosis is considered to be an insidious disease due to the lack of obvious progressive manifestations. It is also called a “silent” disease, because osteoporosis often shows no manifestations until an osteoporotic fracture occurs (Rachner et al., 2011; Dhillon, 2016). Zoledronic acid (ZA), a nitrogen-containing bisphosphonate, is currently recognized as the first-line pharmacologic treatment for osteoporosis, which can selectively suppress osteoclastic bone resorption through effectively inhibiting farnesyl pyrophosphate synthase (FPPS) activity in the mevalonate pathway (Black et al., 2007; Rogers et al., 2020).

Osteoclasts arise by the fusion of precursors derived from hematopoietic stem cell populations in the bone marrow, and are mainly responsible for the resorption of old bone (Fierro et al., 2017; Jacome-Galarza et al., 2019; Muruganandan et al., 2020; Kameda et al., 2021). There is a dynamic balance between the old bone resorption by osteoclasts and new bone formation by osteoblasts in order to maintain the stability of bone tissue in the body (Rachner et al., 2011; Muruganandan et al., 2017; Chen et al., 2018; Muruganandan et al., 2018). Metabolic bone diseases, e.g. osteoporosis, occur when this balance is disrupted due to some reasons (Rachner et al., 2011; Dhillon, 2016; Ryoo et al., 2020). Osteoporosis is mainly caused by osteoclast-induced bone resorption. Active osteoclasts can break down and absorb bone rapidly, resulting in a decrease reduction in bone mass, an increase in bone voids, and causing osteoporosis. The radiographic features include bone rarefaction, decreased bone mass, disruption of trabecular continuity. The occurrence and development of osteoporosis are closely related to osteoclasts, so the key for treatment of osteoporosis is to focus primarily on inhibiting osteoclast differentiation and inducing osteoclast apoptosis (Tan et al., 2017; Da et al., 2021; Qiu et al., 2022; Wang et al., 2022).

ZA is a third-generation bisphosphonate with high affinity for bone tissues, which exerts potent anti-resorptive activity through multiple pathways, and simultaneously inhibits osteoclast differentiation and induces osteoclast apoptosis. (Lu et al., 2020; Wang et al., 2020; Lin et al., 2021; Liu et al., 2021; Qu et al., 2021). Therefore, in this review, we intend to summarize the latest research progress on the mechanisms of ZA in inhibiting osteoclast differentiation and inducing osteoclast apoptosis, with the hope of providing new strategies and ideas for the treatment of osteoporosis.

## Zoledronic acid inhibits osteoclast differentiation

The receptor activator of the nuclear factor-κB ligand (RANKL)/the receptor activator of the nuclear factor-κB(RANK) signaling pathway plays a pivotal role in osteoclast differentiation.

RANK was discovered by Anderson et al. (Anderson et al., 1997) when they were analyzing the cDNA sequence of dendritic cells. RANK is a type I homotrimeric transmembrane protein composed of 616 amino acids, and it is the only known RANKL receptor agonist (Canullo et al., 2016). The joint action of RANK and RANKL can promote the differentiation and formation of osteoclasts and osteoclast precursor cells, thereby promoting the differentiation and formation of osteoclasts. At the same time, the joint action of RANK and RANKL can not only promote the differentiation and formation of osteoclasts, it can also inhibit the metabolism and apoptosis of osteoclasts (Anderson et al., 1997; Ikebuchi et al., 2018).

RANKL belongs to the tumor necrosis factor (TNF) family and can also be called osteoclast differentiation factor. RANKL is a type II homotrimeric protein, which is considered to be essential in the process of osteoclast activation and proliferation. It is a key factor affecting the differentiation and formation of osteoclasts and the formation of bone resorption and bone formation (Kiesel and Kohl, 2016). RANKL was found to have three different subtypes, RANKL 1, RANKL 2, and RANKL3. Although these three subtypes are slightly different in structure, they all can promote the proliferation and differentiation of osteoclasts, thereby affecting bone resorption and bone formation (Piemontese et al., 2016). Xu et al. (Xu et al., 2016) believed that osteoclast differentiation could be induced by adjusting the ratio of RANKL/OPG. Liu et al. (Liu et al., 2016) considered the OPG/RANKL interaction as a mechanism to maintain bone homeostasis in their studies. Abdelmagid et al. (Abdelmagid et al., 2015) found in their experiments that mutated osteoprotegerin could promote RANKL-mediated osteoclast differentiation and survival, but inhibit osteoclast function. Studies by many scholars have reached the same view that RANK and RANKL play important roles in the differentiation and proliferation of osteoclasts and the dynamic balance of bone resorption and bone formation.

The RANK/RANKL signaling pathway plays an extremely important role in the differentiation and formation of osteoclasts. By regulating the activation of osteoclasts, it further regulates bone resorption and participates in bone remodeling. The combination of RANKL and RANK enables osteoclast precursor cells to receive signaling molecules, which promote the differentiation of osteoclast precursor cells into osteoclasts (Jacome-Galarza et al., 2019). According to relevant literature reports (Boyle et al., 2003; Rachner et al., 2011; Jacome-Galarza et al., 2019; Kim et al., 2020; McDonald et al., 2021; Udagawa et al., 2021), RANKL can affect the differentiation of osteoclasts through various pathways, such as NF-κB pathway, JNK pathway, Akt pathway, NFATc1 pathway, etc. Most of these mechanisms are called osteoblast-dependent osteoclast differentiation, because RANKL is produced in osteoblasts.

**FIGURE 1:**
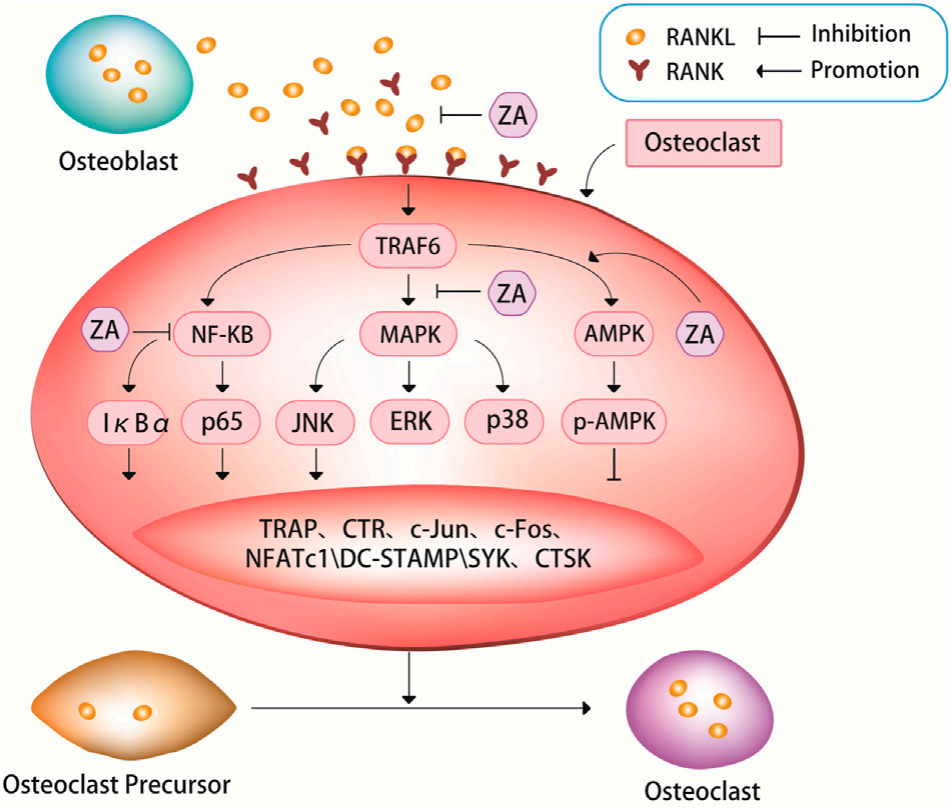
ZA can inhibit the RANKL/RANK signaling pathway. The bind of RANKL and RANK can activate a series of signaling pathways, such as nuclear factor kappa B (NF-κB) pathway, mitogen-activated protein kinases (MAPK) pathway, and AMP-activated protein kinase (AMPK) pathway. These signaling pathways can in turn cause tartrate-resistant acid phosphatase (TRAP), c-Jun, c-Fos, nuclear factor of activation of T cells-1 (NFATc1), c-Jun N-terminal kinase (JNK), dendritic cell Expression of specific transmembrane protein (DC-STAMP), spleen tyrosine kinase (SYK), cathepsin K (CTSK) and other signaling molecules. It is through the RANKL/RANK signaling pathway that ZA regulates the expression of these signaling pathways and signaling molecules to inhibit osteoclast differentiation and bone resorption.

The binding of RANKL to RANK allows the osteoclast precursor cells to receive signaling molecules, and promotes the differentiation of osteoclast precursors into osteoclasts (Boyle et al., 2003; Jacome-Galarza et al., 2019; Kim et al., 2020; McDonald et al., 2021; Udagawa et al., 2021). A study in mice has shown that loss of RANKL can induce severe osteopetrosis, while overexpression of soluble RANKL can lead to severe osteoporosis (Kim et al., 2020). The RANKL/RANK signaling pathway could regulate the survival of osteoclasts through binding of RANKL to its receptor RANK on the surface of osteoclast precursors, and then activate a variety of transcription factors and genes responsible for osteoclast survival and differentiation (Li et al., 2019). ZA can inhibit osteoclast differentiation and bone resorption by suppressing the canonical RANKL/RANK signaling pathway (Figure 1).

ZA can inhibit the fusion of osteoclast precursors and bone resorption activity of osteoclasts by suppressing RANKL/RANK-induced osteoclast-related genes (Cheng et al., 2021; Lin et al., 2021; Huang et al., 2022). It has been demonstrated experimentally that ZA can strongly inhibit the formation of tartrate-resistant acid phosphatase (TRAP)+ multinucleated osteoclasts induced by RANKL/RANK pathway (Cheng et al., 2021). An experimental study treated cells with ZA after 3 days of RANKL stimulation, and found that ZA significantly inhibited the mRNA expression levels of RANK, TRAP and calcitonin receptor in cells (Huang et al., 2022), then the authors further analyzed the effect of ZA on osteoclast-specific markers, the results showed that RANKL stimulation significantly induced the expression of these osteoclast-specific markers, whereas ZA treatment significantly inhibited the expression levels of these markers.

The nuclear factor-kappaB (NF-κB) pathway plays an important role in RANKL-induced osteoclastogenesis (Boyle et al., 2003; Cheng et al., 2021; Zhu et al., 2021). TRAF6 (TNF receptor associated factor 6) is a ubiquitin ligase, when it activated, it can produces short protein chains. So, it can act as a switch to decide what kind of signal to turn on inside the cell. When RANK binds to TRAF6, it activates the NF-κB-inducible kinase, allowing the NF-κB complex to enter the nucleus from the cytoplasm, increasing the expression of c-Fox in the nucleus and binding to activated T cell nuclear factor, inducing osteoclastogenesis genes transcription to promote osteoclast maturation. NF-κB is a family of five transcription factors, including p50, p52, RelA, RelB, and c-Rel. These five transcription factors all have Rel homology domains at their N-termini, which can not only form homologous or heterodimers with each other, but also bind to specific DNA sequences on promoter genes. There are two pathways for the activation of NF-κB: the classical pathway and the non-canonical pathway. RelA and c-Rel preferentially form heterodimers with p50, and RelA/p50 can activate most of the key signaling molecules in the canonical NF-κB pathway. After the activation of the signal molecules, the canonical NF-κB pathway is rapidly activated (Boyce et al., 2015). The non-canonical NF-κB pathway is activated several hours after the initiation of the canonical NF-κB pathway through the nuclear transfer of the RelB/p52 heterodimer. This activation process is slower than that of the classical NF-κB pathway (Dutta et al., 2017). Regardless of which NF-κB pathway, it can affect the activation and proliferation of osteoclasts and bone resorption. IκBα and p65 are inhibitors of NF-κB, which can suppress NF-κB signaling pathway. RANKL can induce IκBα phosphorylation, thereby promoting osteoclast differentiation and bone resorption (Sun et al., 2020; Cheng et al., 2021; Huang et al., 2022). ZA can down-regulate RANKL-induced phosphorylation of IκBα and p65, simultaneously up-regulate the level of non-phosphorylated IκBα, and inhibit the nuclear translocation of p65 (Cheng et al., 2021; Huang et al., 2022). An experimental study showed that rapid phosphorylation of IκBα and p65 was detected in RANKL-treated cells, indicating that the NF-κB pathway was activated, however, after the cells were treated with ZA, the opposite results were obtained, i.e. the level of non-phosphorylated IκBα was significantly elevated, and p65 phosphorylation was attenuated by ZA in a dose-dependent manner (Huang et al., 2022). The same results were also found in an animal study, results from western blot of this experimental study in rats showed that at weeks 4 and 12 after ovariectomy, the expression levels of RANKL, p65, and phosphorylated forms of p65 and IκBα were significantly higher in the ovariectomy group than in the ovariectomy + ZA group (Huang et al., 2022).

Both the mitogen-activated protein kinases (MAPK) and NF-κB signaling pathways are closely related to osteoclast differentiation. The MAPK pathway, including extracellular signal-regulated kinase (ERK), c-Jun N-terminal kinase (JNK), and p38, is critical for regulation of osteoclast differentiation (Jin et al., 2018; Huang et al., 2019; Peng et al., 2021). ZA treatment can down-regulate JNK phosphorylation in a dose-dependent manner. However, ZA cannot significantly affect the phosphorylation levels of p38 and ERK, suggesting that ZA can specifically inhibit RANKL-induced JNK phosphorylation during osteoclast differentiation (Jin et al., 2018; Huang et al., 2019; Sun et al., 2020; Cheng et al., 2021; Huang et al., 2022). ZA can inhibit RANKL-induced downstream expression of c-Jun, c-Fos and nuclear factor of activation of T cells-1 (NFATc1). NFATc1, also known as calcineurin (CN), it can activate nuclear factor of activated T-cells (NFAT) and rapidly enter the nucleus to participate in osteoclast gene expression. NFATc1 is considered to be the most important intracellular regulator of osteoclastogenesis, and RANKL acts by recruiting RelA/p50 and activating T cell nuclear factor 2 protein (NFAT2) to promote the expression of activating T cell nuclear factor c1 (NFATc1). A large amount of NFATc1 can antagonize the signaling molecules that inhibit RANK in the downstream pathway, thereby promoting the growth and differentiation of osteoclasts. A experimental study showed that the expression of c-Jun, c-Fos and NFATc1 was significantly increased in cells after culturing with RANKL, but this increase was suppressed after subsequent treatment with ZA, ultimately leading to the inhibition of osteoclast differentiation and maturation, and decrease in osteoclastic bone resorption function (Zhao et al., 2021; Huang et al., 2022). Some experimental evidence suggests that the inhibitory effect of ZA on RANKL-induced osteoclast differentiation and bone resorption may be due in part to its inhibition of NF-κB signaling. (Huang et al., 2019; Cheng et al., 2021; Zhao et al., 2021; Huang et al., 2022).

Dendritic cell specific transmembrane protein (DC-STAMP) is known as a key regulator of osteoclast differentiation and fusion. Several recent studies have explored in detail the molecular mechanism of action of DC-STAMP on osteoclast differentiation. During the early stage of osteoclast precursor cell differentiation, DC-STAMP on their surface may be internalized, which induces its mRNA expression and promotes osteoclast fusion (Mullin et al., 2019; Kodama and Kaito, 2020; Søe, 2020). A recent study found that ZA significantly inhibited both protein and mRNA expression of RANKL-induced DC-STAMP (Huang et al., 2022), suggesting that ZA exerts an inhibitory effect on RANKL-induced DC-STAMP expression, which further inhibits RANKL-induced osteoclast differentiation and bone resorption.

A study has shown that AMP-activated protein kinase (AMPK) plays a negative regulatory role in osteoclast differentiation and function (Tong et al., 2020). Multiple drugs can affect osteoclast differentiation and bone resorption through AMPK-mediated signaling pathways, such as AMPK/NF-κB/ERK, AMPK/mTOR/p70S6K, and AMPK/Nfr2 signaling pathways (Li Z et al., 2018; Tong et al., 2018; Peng et al., 2019; Zhao et al., 2020; Guo et al., 2021). Unlike various other drugs whose effects on osteoclast differentiation and bone resorption through AMPK pathway have been widely studied, studies on how ZA affects osteoclasts through the AMPK pathway are relatively recent. A recent study confirmed that after culture of cells with ZA during osteoclastogenesis assay, the expression of AMPK and p-AMPK was increased, and the expression of NFATc1, spleen tyrosine kinase (SYK), cathepsin K (CTSK) and TRAP in cells was decreased, additionally, osteoclast differentiation and bone resorption were inhibited (Dong et al., 2018). However, only one recent study that supports these findings appears to be somewhat underpowered. At present, it is only known that ZA may influence osteoclast differentiation and bone resorption *via* AMPK pathway, but it is still unclear how the AMPK pathway is involved, which need to be further corroborated by more studies. This may be a new target for future research of ZA and osteoporosis.

The results of an experimental study have demonstrated for the first time that ZA may affect osteoclast viability by increasing the protein stability of p53 (Qu et al., 2021). This result may suggest that there is a link between ZA and p53, ZA can affect osteoclast viability *via* this link. This could be another potential pathway of ZA against osteoclastogenesis, but the exact mechanism is not yet clear.

The Wnt signaling pathway is another pathway that has a large impact on osteoclast differentiation. The Wnt signaling pathway can be divided into two categories: canonical and non-canonical. The canonical Wnt signaling pathway promotes bone formation, while the non-canonical Wnt/Ca2+/CaMKII pathway is involved in mediating osteoclast differentiation (Karner and Long, 2017; Zhang et al., 2018; Maeda et al., 2019; Wang et al., 2020). After stimulation of osteoclasts, intracellular Ca2+ concentration is up-regulated in cells, which in turn activates the binding of calmodulin to CaMKII, promotes the expression of NFATc1 and TRAP, eventually inducing osteoclast differentiation (Maeda et al., 2019). ZA can decrease the protein levels of non-canonical Wnt proteins, Wnt5a and CaMKII, and the final result is the obviously inhibition of osteoclast differentiation (Zhang et al., 2018).

Macrophage colony stimulating factor (M-CSF) is also known as colony stimulating factor-1 (CSF-1). M-CSF is a homodimeric glycoprotein, a cytokine that can be secreted and expressed by osteoblasts, stromal cells and T lymphocytes. (Yang et al., 2017; Győri and Mócsai, 2020). For example, c-fms is a tyrosine kinase receptor, which can specifically bind to M-CSF. After c-fms binds to M-CSF, it can activate the tyrosine kinase activity of c-fms, resulting in its own phosphorylation and decomposition for c-src, phosphatidylinositol kinase 3-kinase (PI3K), Grb2, phosphorylated c-fms binds with Grb2 to ERK to promote osteoclast differentiation (Kim and Kim, 2016). Kim et al. (Kim et al., 2016) have shown that M-CSF can also inhibit osteoclast apoptosis, promote the combination of RANKL and RANK on the surface of osteoclasts, improve the sensitivity of RANK to RANKL, and promote osteoclast differentiation. According to the existing research, many scholars believe that the differentiation of osteoclasts can only occur under the co-stimulation of RANKL and M-CSF. The combined application of M-CSF and RANKL can replace the effect of osteoclasts on the differentiation of monocytes and macrophages into osteoclasts. In addition, the ERK, Akt, and c-fos signaling pathways can co-stimulate RANKL and M-CSF to promote the differentiation and maturation of osteoclasts (Yang et al., 2017; Ono and Nakashima, 2018; Győri and Mócsai, 2020). ZA inhibits the activity, aggregation and migration of osteoclast precursor cells and macrophages to prevent osteoclast differentiation and induce apoptosis (Morii et al., 2015; Li M et al., 2018; Shibuya et al., 2019; Huang et al., 2022). Studies have shown that in the presence of M-CSF and ZA can also inhibit RANKL-induced upregulation of RANK mRNA, thereby inhibiting osteoclast differentiation (Kimachi et al., 2011). However, there is currently insufficient research evidence to directly demonstrate the relationship between ZA and M-CSF, but this may be another potential pathway by which ZA inhibits osteoclast proliferation and differentiation and counteracts bone resorption.

## Zoledronic acid induces apoptosis in osteoclasts

**FIGURE 2:**
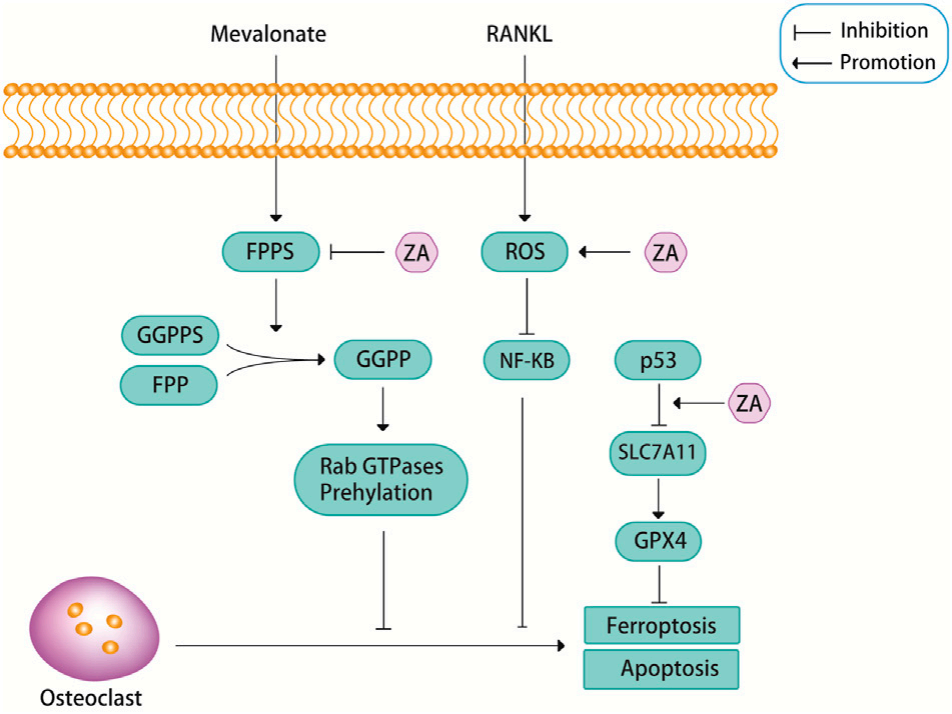
ZA can inhibit the mevalonate pathway. Farnesyl pyrophosphate synthase (FPPS), as a key regulatory enzyme in the mevalonate pathway, can promote the formation of geranylgeranyl diphosphate (GGPP) from geranyl pyrophosphate synthase (GGPPS) and farnesyl diphosphate (FPP), and GGPP can promote the prenylation of Rab GTPases, this pathway is closely related to the apoptosis of osteoclasts. ZA promotes osteoclast apoptosis by inhibiting the activity of FPPS. In addition, ZA can promote osteoclast apoptosis through reactive oxygen species (ROS). Recent findings suggest that ZA may promote ferroptosis in osteoclasts through p53.

FPPS is a key enzyme in sterol metabolism and is located at the intersection of different pathways, including the pathways that are involved in the biosynthesis of isoprenoids, dolichols, ubiquinones and ergosterol/cholesterol (Gadelha et al., 2020). FPPS is a key enzyme in the mevalonate pathway, which can promote osteoblast differentiation by catalyzing the synthesis of geranyl pyrophosphate (GPP) and farnesyl diphosphate (FPP). FPP can be converted into geranylgeranyl diphosphate (GGPP) through catalyzing by geranylgeranyl diphosphate synthase (GGPPS) (Tang et al., 2017; Malwal et al., 2018; Gadelha et al., 2020; Park et al., 2021). Excessive amounts of FPP and GGPP lead to protein prenylation impairment, this is a potential pathological feature of osteoporosis (Zameer et al., 2018). ZA is known as the gold standard for the treatment of osteoporosis because of its recognized role in attenuating bone resorption and osteoclast apoptosis by inhibiting FPPS in the mevalonate pathway (Figure 2) (Tang et al., 2017; Zameer et al., 2018; Chandra and Rajawat, 2021; Chen et al., 2021; Munoz et al., 2021). Targeted inhibition of FPPS by ZA blocks the synthesis of FPP and the downstream product GGPP, which prevents the prenylation of Rab GTPases (e.g., members of the Ras superfamily) and their binding proteins (Kimachi et al., 2011; Zameer et al., 2018; Soysa and Alles, 2019). The prenylation of Rab GTPases and their binding proteins is crucial for osteoclast survival, so this blocking action of ZA induces osteoblast apoptosis.

Reactive oxygen species (ROS), including superoxide anion (O2-), hydrogen peroxide (H2O2), and nitric oxide (NO), are essential for cell signaling and other physiological functions (Tai et al., 2017; Agidigbi and Kim, 2019). However, excessive ROS can cause cellular imbalance in reduction-oxidation reactions and disrupt normal biological functions, resulting in oxidative stress, a condition known to contribute to the development of various diseases, such as osteoporosis (Tai et al., 2017; Agidigbi and Kim, 2019). ROS are important components that regulate osteoclast differentiation. Osteoclasts are very sensitive to oxidative stress, and low levels of ROS may stimulate osteoclast bone resorption during bone resorption and osteoclast differentiation (Tai et al., 2017). However, when ROS levels exceed a certain threshold, prolonged exposure of osteoclasts to elevated oxidative stress can lead to cytotoxic effects due to increased oxidative damage of DNA, proteins and lipids, which can then lead to apoptosis *via* the caspase-dependent pathway (Tai et al., 2017). A study showed that ZA can cause PI3K/AKT inactivation, glycogen synthase kinase (GSK)-3β activation, and myeloid cell leukemia 1 (Mcl-1) down-regulation through inducing NADPH oxidase-mediated ROS production, thereby further inducing apoptosis in osteoclast precursors (Figure 2) (Tai et al., 2017). There is an important relationship between the PI3K/AKT signaling pathway and osteoclast differentiation, survival and the interaction of various cytokines. An experimental study found that ZA inhibited the activation of the PI3K/AKT signaling pathway, this may also be the reason for ZA to induce osteoclast apoptosis and reduce bone destruction (Liu et al., 2021).

Ferroptosis is a newly discovered iron-mediated cell death, is often accompanied by massive iron accumulation and lipid peroxidation during the cell death process (Hirschhorn and Stockwell, 2019; Li et al., 2020; Qu et al., 2021). Glutathione peroxidase 4 (GPX4) is a core regulatory protein of ferroptosis that can use glutathione to protect cells from ferroptosis by eliminating phospholipid peroxides (Yang and Stockwell, 2016). A recent study found that ZA inhibited osteoclast viability in a dose-dependent manner, and the authors demonstrated that ZA promoted ferroptosis in osteoclasts by increasing the protein stability of p53 (Figure 2) (Qu et al., 2021). But in fact, p53 has been found to promote ferroptosis by inhibiting the expression of SLC7A11 and affecting the synthesis of glutathione. So, perhaps the relationship between p53 and ferroptosis is caused by p53 inhibiting the expression of SLC7A11, which in turn leads to a decrease in the activity of GPX4 (Stockwell et al., 2020). Whether the link between ZA and ferroptosis through p53 is also achieved through this signaling pathway is still unclear, and more research is needed to prove it. Another study also showed that ferroptosis was associated with RANKL-induced osteoclast differentiation, which was induced by iron-starvation response and ferritinophagy (Ni et al., 2021). However, there are no definitive answers about whether ferroptosis is involved in ZA-induced osteoclast apoptosis. Perhaps more future studies will be focused on this aspect.

## New directions and hot spots in research of zoledronic acid

Osteoporosis and osteoporosis-related fractures are common causes of morbidity and mortality in the elderly. Due to the changes in population demography, osteoporosis is becoming an increasing worldwide burden on health care systems (Brown, 2017; Eastell and Szulc, 2017; Kendler et al., 2018; Aspray and Hill, 2019; Johnston and Dagar, 2020). Bisphosphonates are the first-line therapy for osteoporosis, and ZA is a typical representative of bisphosphonates (Johnston and Dagar, 2020). Among the bisphosphonates, ZA has been shown to have better persistence, and once-yearly intravenous injections is effective in improving osteoporosis (Fobelo Lozano and Sánchez-Fidalgo, 2019). ZA is helpful to alleviate clinical symptoms, reduce the degree of bone pain, promote the increase of bone mass, and has a high safety for the treatment of senile osteoporosis, which is the favored treatment for osteoporosis (Kong et al., 2020). The excellent anti-osteoporosis effect of ZA is mainly attributed to its ability to specifically inhibit osteoclast-mediated bone resorption and osteoclast activation, and even act directly on osteoclasts to inhibit their formation, thereby accelerating osteoclast apoptosis and increasing bone mineral density (Fobelo Lozano and Sánchez-Fidalgo, 2019; Kong et al., 2020). There have been years of research on the efficacy of ZA in the treatment of osteoporosis *via* regulating osteoclasts. The current mainstream focus have been still on the inhibitory effect of ZA on osteoclast differentiation through the RANKL signaling pathway (Li et al., 2019; Kim et al., 2020). The classical RANKL/RANK signaling pathway is the earliest and most widely studied signaling pathway for osteoclast differentiation, and RANKL is the ligand required for osteoclastogenesis, so studies of osteoclast differentiation have always revolved around the RANKL signaling pathway (Yasuda, 2021). Therefore, how ZA regulates osteoclast differentiation through the RANKL pathway has always been a research hot spot. Through reviewing recent studies, it is not difficult to see that ZA exerts its regulatory effect on osteoclast differentiation through the entire RANKL signaling pathway, which can target many classical regulators or osteoclast differentiation-associated genes such as RANK, JNK, c-Jun, c-Fos and NFATc1 and DC-STAMP (Huang et al., 2019; Mullin et al., 2019; Kodama and Kaito, 2020; Søe, 2020; Cheng et al., 2021; Peng et al., 2021; Huang et al., 2022). The non-canonical Wnt/Ca2+/CaMKII signaling pathway is another pathway by which ZA regulates osteoclast differentiation (Wang et al., 2020). ZA can not only inhibit the differentiation, but also induce the apoptosis of osteoclasts. FPPS, ROS and ferroptosis, a newly discovered type of cell death, are currently the focuses of the studies on inhibition of osteoclast differentiation by ZA (Tai et al., 2017; Zameer et al., 2018; Ni et al., 2021; Qu et al., 2021). Ferroptosis, in particular, has been shown to be closely related to the occurrence of various diseases, such as cancer and neurological diseases (Xu et al., 2019). A previous study has attempted to demonstrate the relationship between ferroptosis and osteoporosis (Hadian and Stockwell, 2020; Qiu et al., 2020). Recently, study on how ZA induces osteoclast apoptosis through ferroptosis has also been conducted (Qu et al., 2021). Undoubtedly, this has provided a new way for studying ZA and osteoclasts.

At present, ZA is not just an anti-osteoporosis drug, many studies have reported the link between osteoclasts and cancer (Finianos and Aragon-Ching, 2019; Liu et al., 2019; Boran et al., 2020). Skeletal-related events (SREs) occur frequently in cancer bone metastases, and in some studies of zoledronic acid and SREs, ZA has been found to be effective in preventing SRE, reducing pain, and improving quality of life (Mhaskar and Djulbegovic, 2018; Miller et al., 2018; Finianos and Aragon-Ching, 2019; Sun et al., 2019; Hu et al., 2020). The link between ZA and osteoclasts was originally studied for the treatment of osteoporosis, now many monoclonal antibodies have been developed to treat osteoporosis, which can replace ZA (Anagnostis et al., 2017; Raje et al., 2018; Makras et al., 2021; Zullo et al., 2021). However, ZA, the longest used and most effective anti-osteoporosis drug, will still be used clinically and studied by researchers and clinicians for a long time in the future.

As a first-line drug for osteoporosis, ZA brings not only benefits, but also side effects. In recent years, it has become increasingly recognized that bisphosphonate use is associated with the incidence of medicine-related osteonecrosis of the jaw (MRONJ) (Beth-Tasdogan et al., 2017; Tamari et al., 2019; Aguirre et al., 2021). ZA is the most potent *in vivo* and is the most commonly used intravenous bisphosphonate with the longest duration of action, so it is associated with the highest risk of MRONJ development (Fliefel et al., 2019). The current research on the relationship between ZA and MRONJ is not very clear. The existing research can only show that ZA and MRONJ may be a multifactorial process, and more research is still needed to unravel the mystery (Ferreira et al., 2020). The side effects of ZA should also be considered in future studies on ZA.

## Conclusion

The tightly regulated balance between bone resorption and bone formation can be altered under pathological conditions. Enhanced maturation and activation of osteoclasts leads to pathological bone resorption, as is common in osteoporosis and inflammatory bone diseases. Therefore, it is of great significance to further explore the related mechanisms of osteoclast differentiation, maturation and functional activity. Osteoclasts are differentiated and formed by the co-regulation of many cell signaling pathways and cytokines, including many canonical and non-canonical signaling pathways.

ZA is currently the first-line drug and gold standard for the treatment of osteoporosis, which can inhibit bone resorption by regulating osteoclast differentiation and apoptosis to achieve anti-osteoporosis effect. How ZA regulates osteoclast differentiation and apoptosis has been studied for many years, which is still a research hot spot to date. In this paper, we summarize and discuss the latest progress on the research of ZA and osteoclasts, highlighted the relationship between canonical RANKL pathway, non-canonical Wnt pathway, M-CSF, the inhibition of osteoclast differentiation by ZA, as well as the relationship between ROS, ferroptosis and the induction of osteoclast apoptosis by ZA. As the current classic first-line drug, ZA has excellent anti-osteoporosis effect. There are many cell signaling pathways that regulate osteoclast differentiation. Although the research on ZA and osteoclast differentiation has been carried out for many years, there is still a large research gap. More research on the relationship between ZA and osteoclast differentiation, and exploring the mechanism of ZA have great significance for the future treatment of osteoporosis with ZA and even newer drugs.
